# Specialisation of spinal services: consequences for cervical trauma management in the district hospital

**DOI:** 10.1186/1752-2897-1-6

**Published:** 2007-11-30

**Authors:** Ulfin Rethnam, James Cordell-Smith, Amit Sinha

**Affiliations:** 1 Department of Orthopaedics, Glan Clwyd Hospital, Bodelwyddan, UK; 2 Department of Orthopaedics, Morriston Hospital, Swansea, UK

## Abstract

**Background:**

Specialisation in spinal services has lead to a low threshold for referral of cervical spine injuries from district general hospitals. We aim to assess the capability of a district general hospital in providing the halo vest device and the expertise available in applying the device for unstable cervical spine injuries prior to transfer to a referral centre.

**Methods:**

The study was a postal questionnaire survey of trauma consultants at district general hospitals without on-site spinal units in the United Kingdom. Seventy institutions were selected randomly from an electronic NHS directory. We posed seven questions on the local availability, expertise and training with halo vest application, and transferral policies in patients with spinal trauma.

**Results:**

The response rate was 51/70 (73%). Nineteen of the hospitals (37%) did not stock the halo vest device. Also, one third of the participants (18/51, 35%, 95% confidence interval 22 – 50%) were not confident in application of the halo vest device and resorted to transfer of patients to referral centres without halo immobilization.

**Conclusion:**

The lack of equipment and expertise to apply the halo vest device for unstable cervical spine injuries is highlighted in this study. Training of all trauma surgeons in the application of the halo device would overcome this deficiency.

## Background

In the United Kingdom (UK), most spinal trauma presents to district general hospitals where on-site spinal units are unavailable. Patients need to be transferred to tertiary care centres for definitive surgical management.

Unstable cervical spine injuries require adequate immobilisation to prevent or limit neurological sequelae during transport. Methods of immobilisation of the injured cervical spine include cervical orthotics (hard cervical collar), head cervical orthotics (Philadelphia collar and Miami-J collar), cervical traction, and halo-vest immobilisation. The halo vest is the most rigid of all cervical orthoses [1], and represents the immobilisation method of choice when preparing patients for transfer between hospitals [2].

Although this is an effective and relatively safe procedure [3], Kang and co-workers felt that familiarity with the design, rationale of usage, proper method of application, and awareness of potential complications could minimize the morbidity associated with the use of the halo vest device [4].

We investigated the capability of UK district general hospitals regarding the familiarity and confidence of application of the halo vest traction device among the orthopaedic staff, availability of the device, and the implications this may have for training and service delivery in light of the ongoing restructuring of spinal services towards tertiary spine centres.

## Methods

A survey was conducted at 70 UK district general hospitals with designated acute trauma admission status. Eligible centres were identified randomly using an electronic NHS directory.

Hospitals with on-site spinal units were excluded. Individual orthopaedic trauma consultants were contacted by a postal questionnaire to assess the level of service provision with regard to halo vest application.

The questionnaire was in a simple tick-box style format and assessed whether the hospitals in which the consultants were employed stocked halo vest equipment routinely, their level of confidence to apply halo devices to adult and paediatric trauma patients, and whether they had received adequate training in application or had recent experience in halo vest application.

In addition, participants were asked about referral protocols and problems encountered with referral of patients with cervical spine injuries to tertiary spine centres.

Results are presented as absolute numbers and proportions together with 95% binomial exact confidence intervals (CI), where appropriate.

## Results

Altogether, 51/70 consultants responded to the questionnaire, for a response rate of 73%.

Nineteen (37%) of 51 district hospitals no longer routinely stocked emergency halo-vest equipment. Just 33/51 (65%, 95% CI 50 – 78%) of the consultants stated that they would feel confident to apply this device even when available both in adults and children, while the remaining did not feel confident either because of inadequate training or lack of recent experience.

Twenty consultants (39%, 95% CI 26 – 54%) did not receive adequate training in applying the halo vest device. Only fifteen (29%, 95% CI 17 – 44%) had applied a halo vest in the past two years.

Most surgeons had a low threshold in referring patients to tertiary spinal units despite inherent risks associated with transfer of an unstable cervical injury with suboptimal immobilisation (34/51, 67%, 95% CI 52 – 79%). This was despite one quarter of clinicians (12/51, 24%, 95% CI 13 – 37%) encountered referral difficulties such as inappropriate delays or problems obtaining specialist advice.

## Discussion

Cervical spine injuries can have serious neurological consequences. Patients with these injuries require adequate immobilisation to prevent or limit neurological deterioration during transfer to tertiary spine centres and definitive surgical fixation.

The key factor in immobilising the cervical spine is the rigidity of the applied device. Cervical and head-cervical orthoses still allow for variable motion of the cervical segments and therefore are not suitable in patients with unstable cervical spine injuries. Studies assessing the stabilising effects of different cervical orthoses showed the halo-vest device to be the most rigid [5,6].

The treatment of unstable cervical spine injuries with the halo vest is an established procedure. The halo traction device was first devised by Perry and Nickel in 1959 to overcome problems encountered while using the Minerva plaster for treating unstable cervical spine fractures [7]. The halo traction device provides good control of flexion, extension and rotation of the upper cervical spine [8,7].

The halo vest can be used for both intermediate and definitive treatment of cervical spine injuries, as well as immobilisation after surgical fixation of cervical spine fractures [9]. It may even be used for treatment of unstable cervical and upper thoracic fractures and dislocations as low as Th 3.

The halo ring is made of graphite or metal with pin fixation on the frontal and parieto-occipital areas of the skull. Development of lightweight composite material led to the design of radiolucent rings compatible with magnetic resonance imaging. Restriction in cervical motion depends on the fit of the halo vest, since improper fit can allow 31% of normal spine motion. The halo vest is the weak link in terms of motion control. Compressive and distractive force can occur with variable fit of the vest. Motion restrictions provided by the halo include the following: limits flexion and extension by 90 to 96%, limits lateral bending by 92 to 96%, limits rotation by 98 to 99% [4].

When compared to cervical traction using skull tongs the halo-vest device keeps patients mobile and reduces respiratory problems. This is specifically advantageous in elderly patients who have a higher incidence of upper cervical spine injuries [10].

Despite its efficacy in immobilising the cervical spine, the halo vest device has its own problems. Complications like pin loosening, pin site infection, discomfort at pin sites, dysphagia, prolonged bleeding at pin sites, and dural puncture have been reported in the literature [1]. This can be reduced by familiarity with the design, and awareness of proper method of application.

Although in the UK most spinal trauma cases present initially to district general hospitals, our study shows a trend not to stock the halo device in one third of these hospitals. This would mean immobilisation of potentially unstable cervical spine injuries by other, less rigid cervical orthoses. When the halo device was available, only two thirds of the trauma surgeons were confident in applying one. Previously, this would have been considered a prerequisite trauma skill for practicing orthopaedic surgeons in hospitals providing acute services. There now appears to be a wide variation in the provision of this essential service throughout the UK, with a high proportion of trauma units having neither the resources nor clinical expertise to manage these injuries. As the management of spinal trauma becomes more specialised, this is likely to affect service delivery and training, and has important safety implications.

One limitation of our study is that, although the sample population was selected randomly, it may still not be representative of all district general hospitals in the UK. Also, the overall sample size and response rate may further limit firm conclusions. Finally, we did not collect data on demographic and professional backgrounds of the respondents and their institutions.

Apart from these limits, our study has created an awareness of the existing level of application skill and availability of the halo vest traction device. No comparable study is available in the literature, and it may be of interest to perform similar surveys in other countries.

We recommend training all trauma surgeons in the indications, technique of application, and possible complications of the halo vest device.

## Conclusion

Specialisation of spinal services has serious implications on the initial management of cervical spine trauma in district general hospitals without on-site spinal units. The lack of equipment and expertise to apply the halo vest device for unstable cervical spine injuries in this set up is highlighted. We recommend training of all trauma surgeons in the application of a halo vest device and making this device available for use.

## Competing interests

The author(s) declare that they have no competing interests.

## Authors' contributions

UR was involved in reviewing the literature, drafting the manuscript and proof read the manuscript. JCS was involved in collecting data, reviewing the literature, drafting the manuscript and proof read the manuscript. AS is the senior author and was responsible for final proof reading of the article. All authors have read and approved the final manuscript.
